# Standardized antimicrobial resistance surveillance in Somaliland: A hospital-based baseline evaluation using CLSI M39 and WHONET

**DOI:** 10.1016/j.nmni.2026.101861

**Published:** 2026-09-30

**Authors:** Nouh Botan, Abdisamad Sh O. Ali, Hamse A. Warsame, Zainab Ali

**Affiliations:** a Hargeisa Group Hospital, Hargeisa, Somaliland, Federal Republic of Somalia; b Independent Public Health Researcher, Hargeisa, Somaliland, Federal Republic of Somalia

**Keywords:** Antimicrobial resistance, WHONET, CLSI M39, Antibiogram, Surveillance, Somaliland, Antimicrobial stewardship, Horn of Africa

## Abstract

**Background:**

Antimicrobial resistance (AMR) surveillance remains limited across the Horn of Africa because of constrained laboratory infrastructure and fragmented microbiological reporting systems. This study established the first hospital-based cumulative antibiogram in Somaliland using Clinical and Laboratory Standards Institute (CLSI) M39-A4 methodology and WHONET software to provide a baseline surveillance model for resource-limited healthcare settings.

**Methods:**

A retrospective surveillance study was conducted using microbiology records collected over a completed 12-month period (January–December 2025) at a tertiary referral hospital in Somaliland. Duplicate isolates were removed according to CLSI M39-A4 recommendations by including only the first isolate per patient per species. Intermediate susceptibility results were categorized as resistant. Subgroup comparisons by specimen source were evaluated using Fisher’s exact test.

**Results:**

From an initial dataset of 286 positive cultures generating 1,787 individual susceptibility records, 277 unique bacterial isolates were analyzed. *Escherichia coli* was the predominant pathogen (50.9%, n = 141), followed by *Staphylococcus aureus* (33.6%, n = 93). For *E. coli*, nitrofurantoin demonstrated the highest susceptibility rate (78.5%), followed by gentamicin (60.4%). Reduced phenotypic susceptibility to meropenem was observed among tested Gram-negative isolates, with 30.5% classified as susceptible. Wound/pus *E. coli* isolates demonstrated lower meropenem susceptibility than urinary isolates (13.3% vs. 35.4%), although subgroup differences did not reach statistical significance (p = 0.128).

**Conclusion:**

This study establishes the first standardized cumulative antibiogram generated in Somaliland using CLSI M39-A4 and WHONET methodology. The findings demonstrate substantial resistance to multiple commonly used antimicrobial agents and highlight the need to strengthen antimicrobial stewardship, microbiological diagnostics, and structured AMR surveillance systems in resource-limited healthcare settings.

## 1 Introduction

Antimicrobial resistance (AMR) is a major global public health challenge that disproportionately affects low- and middle-income countries (LMICs), where diagnostic capacity, microbiological surveillance systems, and antimicrobial stewardship programs remain limited [1]. The World Health Organization (WHO) has identified AMR surveillance as a critical component for optimizing empirical therapy and monitoring emerging resistance trends through frameworks such as the Global Antimicrobial Resistance and Use Surveillance System (GLASS) [1].

Within the Horn of Africa, implementation of structured AMR surveillance remains inconsistent because of fragmented laboratory infrastructure, limited electronic data systems, restricted culture utilization, and constrained financial resources [2,3]. In Somaliland and neighboring Somali territories, healthcare systems rely heavily on out-of-pocket expenditure, while antimicrobials are frequently obtainable without prescription oversight [2]. Previous regional investigations have also highlighted challenges related to incomplete patient counseling regarding antibiotic dosing intervals and treatment duration, potentially contributing to inappropriate antimicrobial use and selective resistance pressure [4].

Institutional cumulative antibiograms generated according to standardized methodologies provide a practical approach for strengthening antimicrobial stewardship in resource-limited settings [5]. A cumulative antibiogram summarizes the proportion of unique bacterial isolates susceptible to specific antimicrobial agents during a defined surveillance period, assisting clinicians in guiding empirical antimicrobial selection [5,6]. To reduce statistical distortion from repeated isolates obtained from the same patient, the Clinical and Laboratory Standards Institute (CLSI) M39-A4 guideline recommends inclusion of only the first unique isolate per patient per species [5].

Despite increasing microbiology activity within tertiary referral hospitals across East Africa, laboratory data frequently remain underutilized because they are stored in fragmented paper records or isolated spreadsheets without standardized epidemiological analysis [2,7]. Consequently, many healthcare institutions remain unable to generate facility-specific empirical treatment guidance or contribute standardized surveillance data to broader national and international AMR monitoring frameworks [1].

Several studies from Ethiopia and East Africa have reported increasing resistance among Gram-negative organisms to commonly used agents including fluoroquinolones, aminoglycosides, and third-generation cephalosporins [[7], [8], [9], [10]]. Similar resistance profiles have been documented across East African healthcare settings [11,12], reinforcing continental evidence that structured implementation of antimicrobial stewardship and surveillance systems remains highly fragmented across African healthcare contexts [13]. To date, no standardized cumulative antibiogram published according to CLSI M39-A4 guidelines exists for Somaliland, and systematically deduplicated baseline surveillance datasets for the broader Somali territory remain extremely scarce.

This study aimed to establish the first standardized hospital-based cumulative antibiogram in Somaliland using CLSI M39-A4 recommendations and WHONET software. In addition to describing baseline resistance patterns, the study evaluated the feasibility of implementing structured AMR surveillance within a resource-limited tertiary referral healthcare setting.

## 2 Materials and methods

### 2.1 Study design and setting

A retrospective cross-sectional institutional surveillance study was conducted at the principal public tertiary referral hospital in Hargeisa, Somaliland. The facility serves as a major referral center for advanced diagnostic, medical, and surgical services across a broad regional catchment population [2]. Archived routine microbiology and antimicrobial susceptibility testing (AST) records collected over a completed 12-month retrospective period from January 1, 2025, to December 31, 2025, were systematically reviewed. Ethical approval for retrospective laboratory data analysis was obtained from the institutional research committee (Ref: RC-HGHFEBRUARY2026-003).

### 2.2 Data extraction and laboratory practices

Microbiology records were extracted from the laboratory registry and verified during data preparation. The laboratory registry contained routine microbiology diagnostic records generated during the study period. For the purposes of cumulative antibiogram construction, only positive bacterial culture records with antimicrobial susceptibility testing (AST) results were eligible for inclusion. Records representing negative cultures, fungal isolates, and non-bacterial laboratory investigations were excluded during dataset preparation, as they fell outside the scope of bacterial antimicrobial resistance surveillance. This process yielded 286 positive bacterial culture records available for analysis. Because each positive bacterial isolate underwent susceptibility testing against multiple antimicrobial agents, these cultures generated 1,787 individual AST result records within the surveillance database. No additional exclusions for contamination or incomplete records were applied during research data preparation beyond the laboratory's routine diagnostic reporting and validation procedures. Antibiotic-specific denominators varied within the dataset because susceptibility testing reflected routine clinical laboratory practice rather than a prospective, uniform research protocol. This variation includes the use of organism-specific panels, selective testing based on specimen characteristics (e.g., nitrofurantoin applied primarily to urinary isolates), specific clinician requests, and occasional fluctuations in reagent availability. Intermittent shortages of antimicrobial susceptibility disks and other laboratory consumables occasionally influenced the availability of AST for selected antimicrobial agents during the surveillance period. Consequently, not every isolate underwent susceptibility testing against every antimicrobial agent.

### 2.3 WHONET standardization and deduplication

Validated microbiological records were converted into WHONET-compatible format for epidemiological analysis [2]. Following preparation of the analytical bacterial culture dataset, the only additional exclusions applied during cumulative antibiogram construction were 9 repeat patient–organism isolates in accordance with CLSI M39-A4 deduplication guidelines (including only the first unique isolate per patient per species), yielding the final analytic cohort of 277 unique clinical isolates (Fig. 1).

**Fig. 1:**
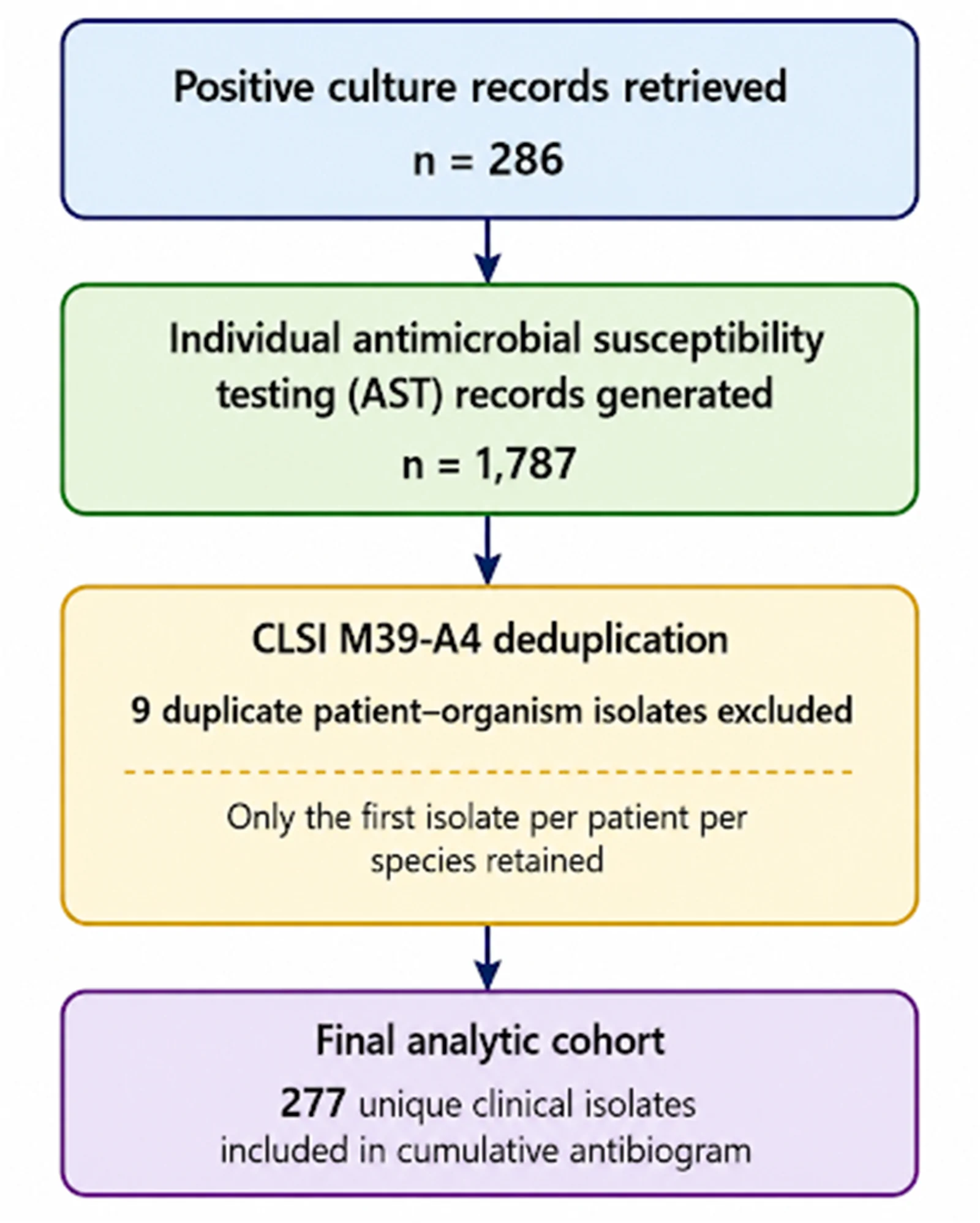
Study flow diagram illustrating the selection of unique clinical isolates from routine laboratory records. Deduplication was performed in accordance with CLSI M39-A4 guidelines, resulting in a final analytic cohort of 277 unique isolates.

Intermediate susceptibility results were categorized as resistant solely for cumulative antibiogram construction in accordance with CLSI M39-A4 recommendations. Individual patient management in routine clinical practice remained based on the laboratory's standard AST reporting procedures.

### 2.4 Statistical analysis

Descriptive statistics were used to summarize pathogen distribution and cumulative susceptibility percentages. Subgroup proportional comparisons between urinary and wound/pus isolates were evaluated using Fisher’s exact test because of small subgroup sample sizes. Statistical significance was defined as a two-tailed p-value <0.05. Data management and visualization were performed using WHONET software integrated with the R statistical computing environment.

## 3 Results

### 3.1 Distribution of bacterial isolates

A total of 277 unique bacterial isolates were included in the final analysis. *Escherichia coli* was the predominant organism, accounting for 50.9% (n = 141) of isolates, followed by *Staphylococcus aureus* at 33.6% (n = 93). *Pseudomonas* species represented 6.9% (n = 19), while other enteric organisms (including *Proteus* spp. and *Klebsiella* spp.) accounted for 8.6% (n = 24) (Fig. 2). A summary of the study population extracted from the laboratory registry is presented in Table 1. The cohort was evenly distributed by sex, with the majority of isolates originating from outpatient services (56.9%). Patient age and detailed clinical diagnostic information were unavailable because the retrospective laboratory database contained only microbiological surveillance variables.

**Fig. 2:**
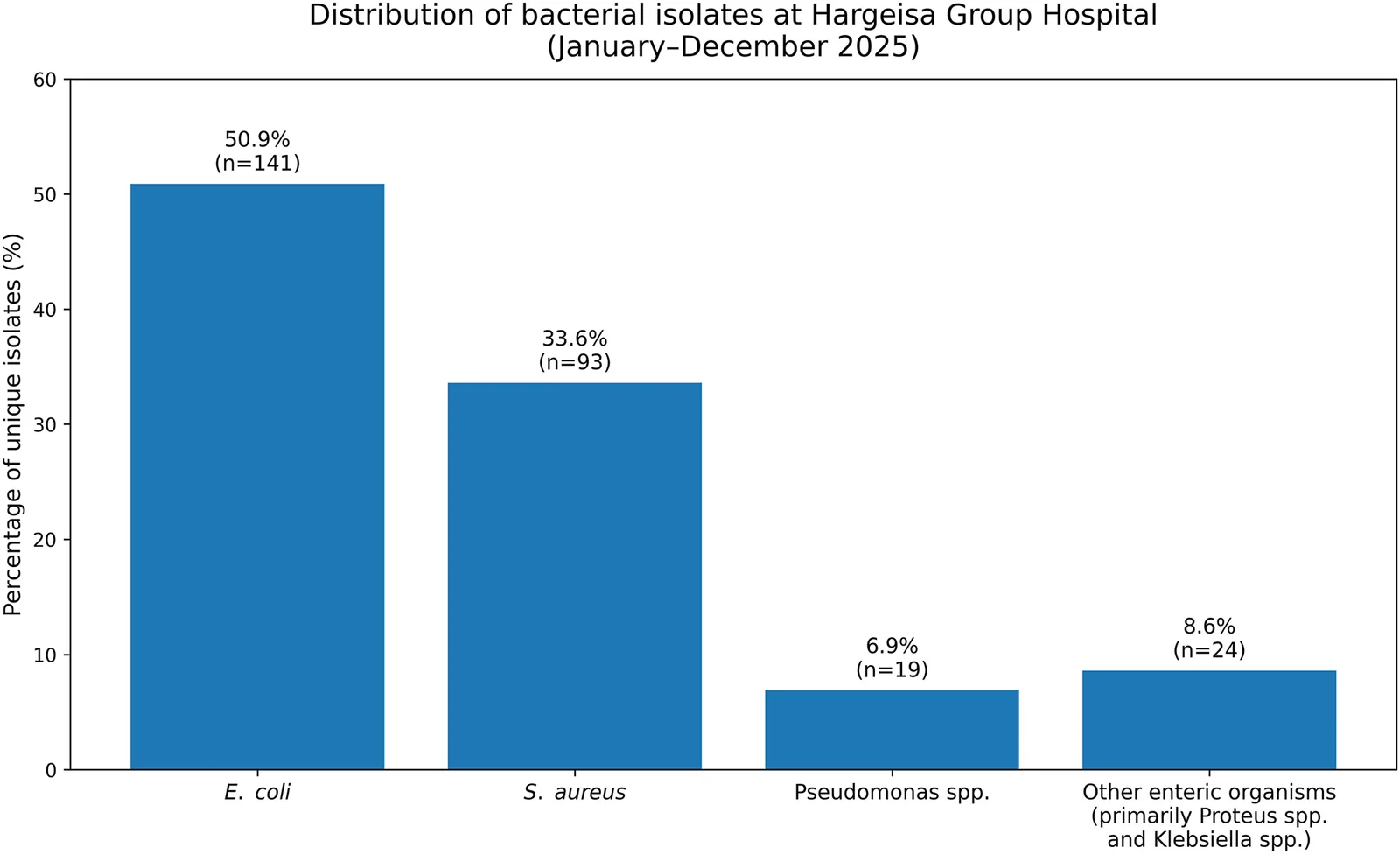
Distribution of bacterial isolates included in the cumulative antibiogram (n = 277) at Hargeisa Group Hospital (January–December 2025). The **other enteric organisms** category primarily comprised *Proteus* spp. and *Klebsiella* spp.; additional organisms were present in smaller numbers.

**Table 1:** Demographic and clinical characteristics of the study cohort (n = 277).

Characteristic	n (%)
**Sex**^a^	
Female	139 (50.4%)
Male	137 (49.6%)
**Ward Source**^a^	
Outpatient	157 (56.9%)
Inpatient	119 (43.1%)
**Specimen Type**	
Urine	140 (50.5%)
Wound/Pus	108 (39.0%)
Blood	6 (2.2%)
Other **	23 (8.3%)

a Data for sex and ward source were missing for 1 isolate. Other includes body fluids, ear swabs, stool, sputum, and throat swabs.

### 3.2 Cumulative antimicrobial susceptibility patterns

The cumulative antimicrobial susceptibility profile for *Escherichia coli* generated across the 12-month surveillance period is presented in Fig. 3 and Table 2. Among legacy frontline agents, ampicillin displayed a low susceptibility rate of just 7.3%. Ciprofloxacin similarly demonstrated highly restricted clinical utility, maintaining a susceptibility rate of only 19.0%, representing an overall resistance burden of 81.0%.

**Fig. 3:**
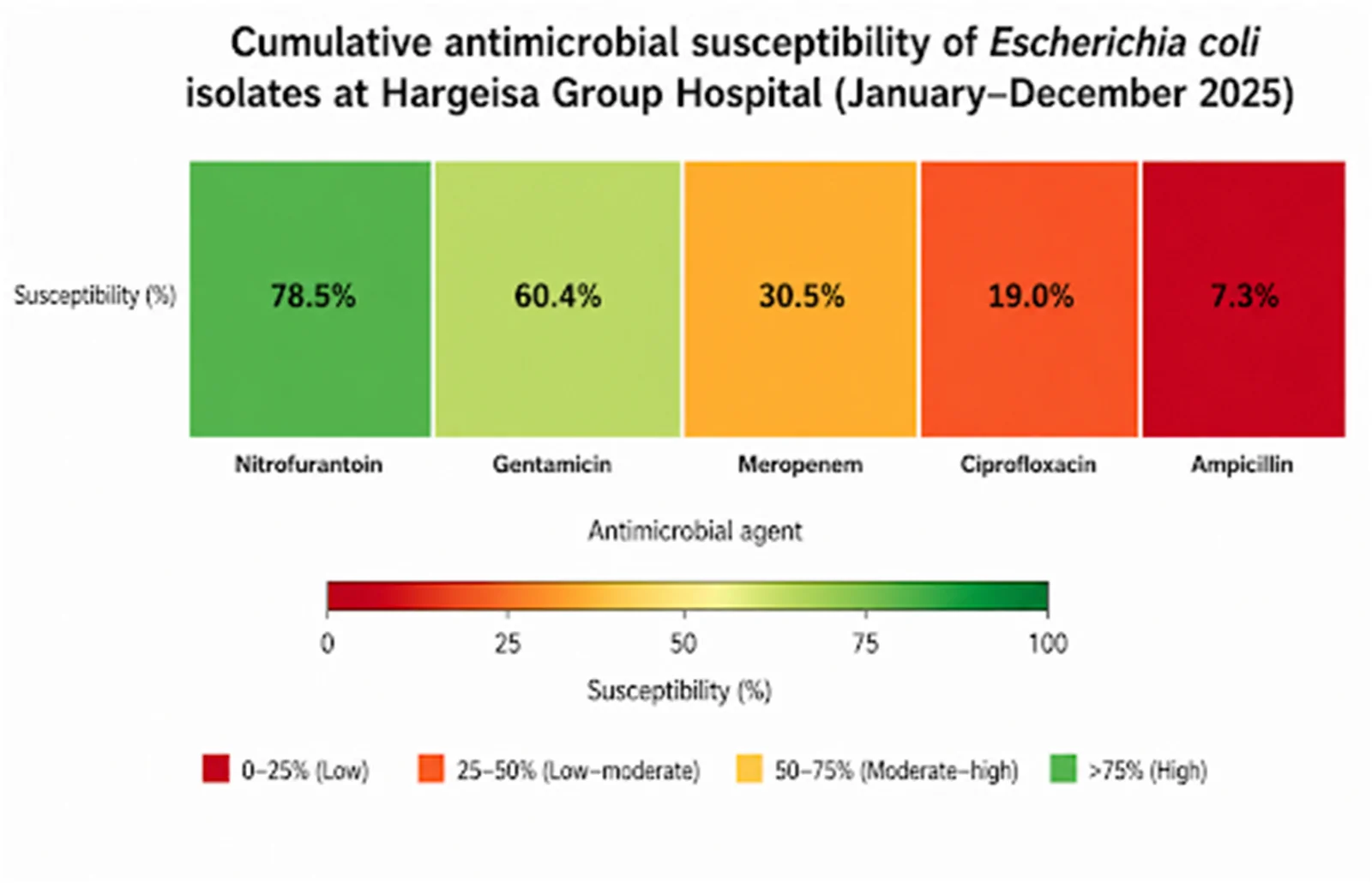
Cumulative antimicrobial susceptibility of *Escherichia coli* isolates at Hargeisa Group Hospital (January–December 2025).

**Table 2:** Organism-specific cumulative susceptibility matrix.

Antibiotic	*E. coli* %S (n tested)	*S. aureus* %S (n tested)	Enterobacteriaceae Pool %S (n tested)	Overall %S
Nitrofurantoin	78.5% (n = 121)	88.3% (n = 77)	76.0% (n = 129)	76.1%
Gentamicin	60.4% (n = 134)	72.1% (n = 86)	51.1% (n = 141)	72.5%
Meropenem	30.5% (n = 82)	Not tested	30.5% (n = 128)	40.0%
Ciprofloxacin	19.0% (n = 79)	26.5% (n = 34)	16.3% (n = 104)	34.1%
Ampicillin	7.3% (n = 82)	Not applicable	7.3% (n = 110)	11.0%

(Note: Antibiotic-specific denominators varied because not every isolate underwent testing against every antimicrobial agent in routine clinical practice).

Conversely, nitrofurantoin remained a consistently reliable therapeutic option within the dataset, maintaining a susceptibility footprint of 78.5% for *E. coli*. Aminoglycoside coverage also provided moderate utility, with *E. coli* demonstrating 60.4% susceptibility to gentamicin. Evaluation of last-line antimicrobial therapy options revealed reduced phenotypic susceptibility to meropenem among tested Gram-negative isolates, with 30.5% classified as susceptible.

### 3.3 Subgroup analysis by specimen source

Comparative subgroup analysis demonstrated variability in antimicrobial susceptibility patterns between urinary and wound/pus *E. coli* isolates (Fig. 4). Wound/pus isolates demonstrated higher ciprofloxacin susceptibility compared with urinary isolates (30.8% vs. 17.2%, p = 0.267), higher gentamicin susceptibility (68.2% vs. 58.7%, p = 0.479), and lower meropenem susceptibility (13.3% vs. 35.4%, p = 0.128). Subgroup analyses were exploratory because of limited sample size; therefore, exact Fisher's exact test p-values are reported and the findings should be interpreted cautiously.

**Fig. 4:**
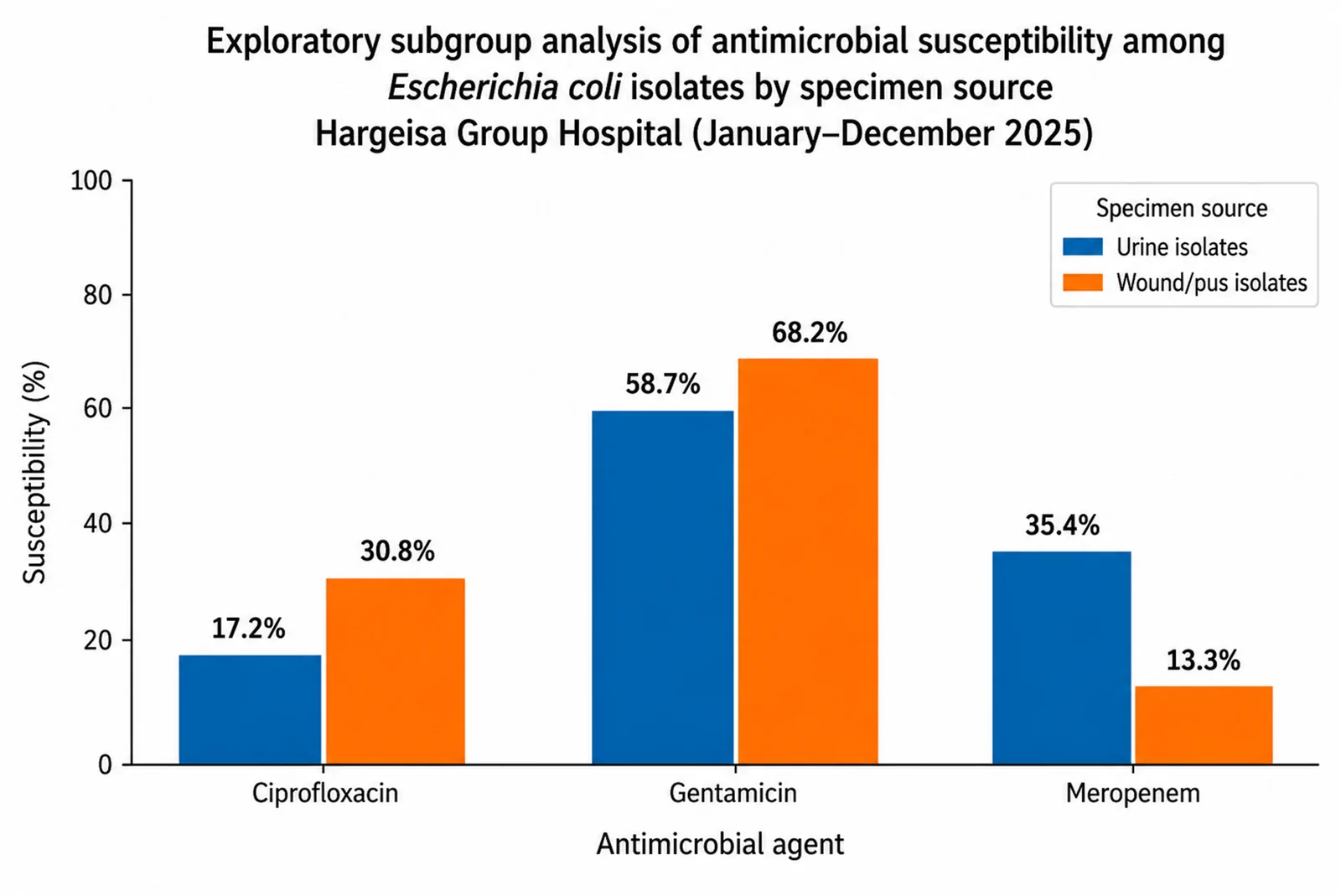
Exploratory subgroup analysis of antimicrobial susceptibility among *Escherichia coli* isolates by specimen source at Hargeisa Group Hospital (January–December 2025).

## 4 Discussion

This study presents the first standardized cumulative antibiogram generated in Somaliland using CLSI M39-A4 methodology and WHONET software. Beyond describing local antimicrobial susceptibility patterns, the study demonstrates the operational feasibility of implementing structured AMR surveillance in a resource-limited healthcare setting currently underrepresented in global surveillance frameworks [2,3].

During the 12-month surveillance period, the laboratory generated 286 positive culture records that were available for cumulative antibiogram analysis. This relatively modest culture volume may have been influenced by broader diagnostic barriers within the local health system. Plausible contributing factors include the out-of-pocket cost of microbiological investigations for patients, reliance on empirical antimicrobial therapy, intermittent laboratory supply constraints, and limited utilization of culture-guided prescribing. Similar challenges affecting microbiological diagnosis and antimicrobial stewardship have been reported across East Africa, where limited laboratory capacity and sparse surveillance data continue to hinder evidence-based antimicrobial prescribing.

The findings revealed low susceptibility rates to several commonly used antimicrobial agents, particularly ampicillin and ciprofloxacin. Similar resistance patterns have been reported across tertiary healthcare facilities in Ethiopia, Kenya, and other East African referral centers [[7], [8], [9], [10]]. Nitrofurantoin maintained comparatively high susceptibility among urinary isolates, supporting its continued role in empirical treatment strategies for uncomplicated urinary tract infections.

The observed reduced phenotypic susceptibility to meropenem among Gram-negative isolates warrants cautious interpretation and should not be interpreted as evidence of specific carbapenemase-producing organisms without species-level identification and molecular confirmation. Given the operational realities of the laboratory, confirmatory molecular characterization and continued prospective surveillance are urgently needed before drawing definitive epidemiological conclusions regarding carbapenem resistance in Somaliland [1,12].

### 4.1 Regional antimicrobial pressure

AMR emergence in resource-limited settings is inherently complex and influenced by broader regional antimicrobial selection pressures. Previous regional literature has documented factors such as unregulated retail pharmaceutical access, veterinary antimicrobial use in pastoral systems, and subsequent environmental exposures [14,15]. Although our primary hospital-based data do not directly investigate these pathways, effective long-term AMR containment strategies will ultimately require integrated collaboration between human healthcare systems, veterinary medicine, and public health surveillance programs to address these systemic drivers [1,3].

### 4.2 Limitations

Several limitations should be acknowledged. First, the study was retrospective and relied on routine laboratory records. Variables that were not consistently available within the retrospective laboratory database (such as patient age or detailed clinical diagnoses) could not be reported. Second, species-level identification beyond routine phenotypic and basic biochemical methods was not consistently available. Advanced diagnostic techniques such as MALDI-TOF MS and molecular characterization were unavailable in this setting. Consequently, some organisms were reported at the genus level in accordance with routine laboratory practice, a limitation that should be considered when interpreting organism-specific resistance patterns. Third, because this study evaluated routine clinical practice, standardized daily internal quality control using ATCC reference strains was not performed because of resource limitations, and the interpretive criteria reflected routine laboratory practice during the study period. Depending on the antimicrobial susceptibility disks available through procurement, breakpoint interpretation followed the manufacturer-recommended CLSI- or EUCAST-based criteria accompanying the respective products. These laboratory practices reflect routine microbiology services in our setting and have now been explicitly acknowledged as methodological limitations. Furthermore, the laboratory procedures described reflect routine clinical practice during the surveillance period and may not be generalizable to other healthcare facilities within Somaliland. Fourth, subgroup analyses were exploratory because of limited sample size. Despite these limitations, strict implementation of CLSI M39-A4 deduplication methodology provides a methodologically standardized baseline for future AMR surveillance efforts in Somaliland [3,5,6].

## 5 Conclusion

This study establishes the first standardized cumulative antibiogram generated in Somaliland using CLSI M39-A4 and WHONET methodology. The findings demonstrate substantial resistance to multiple commonly used antimicrobial agents, including reduced phenotypic susceptibility to meropenem among tested Gram-negative isolates. Strengthening sustainable microbiology laboratory capacity—including the reliable procurement of culture media, antimicrobial susceptibility testing materials, and laboratory equipment—should accompany the implementation of routine cumulative antibiogram surveillance to support antimicrobial stewardship and evidence-based prescribing in Somaliland.

## CRediT authorship contribution statement

**Nouh Botan:** Writing – review & editing, Supervision, Resources, Project administration, Conceptualization. **Abdisamad Sh O. Ali:** Writing – original draft, Software, Methodology, Formal analysis, Conceptualization. **Hamse A. Warsame:** Writing – original draft, Validation, Methodology, Investigation, Data curation. **Zainab Ali:** Writing – review & editing, Writing – original draft, Visualization, Validation, Formal analysis.

## Ethical approval statement

Ethical approval for the retrospective harvesting and analysis of de-identified laboratory surveillance data was reviewed and approved by the Institutional Research Committee of Hargeisa Group Hospital, Somaliland (Reference No. RC-HGHFEBRUARY2026-003). Because this study used anonymized retrospective laboratory records, the requirement for individual informed consent was waived by the approving committee.

## Data availability statement

The anonymized surveillance dataset supporting the findings of this study is available from the corresponding author upon reasonable request for academic research and public health purposes.

## Funding

This research did not receive any specific grant from funding agencies in the public, commercial, or not-for-profit sectors.

## Declaration of competing interest

The authors declare that they have no known competing financial interests or personal relationships that could have appeared to influence or bias the work reported in this paper.
